# 2-(1,3-Benzodioxol-5-yl)-3-phenyl­quinazolin-4(3*H*)-one

**DOI:** 10.1107/S1600536813016346

**Published:** 2013-06-19

**Authors:** Chandra, G. M. Raghavendra, S. Jeyaseelan, K. Mantelingu, M. Mahendra

**Affiliations:** a Department of Studies in Physics, Manasagangotri, University of Mysore, Mysore 570 006, India; b Department of Studies in Chemistry, Manasagangotri, University of Mysore, Mysore 570 006, India; c Department of Physics, St Philomena’s College, Mysore, India

## Abstract

In the mol­ecule of the title compound, C_21_H_14_N_2_O_3_, the quinazoline ring system [maximum deviation = 0.076 (1) Å] makes dihedral angles of 40.57 (9) and 42.31 (11)°, respectively, with the phenyl and 1,3-benzodioxole rings. The dihedral angle between the phenyl ring and the 1,3-benzodioxole ring is 4.34 (10)°. In the crystal, C—H⋯O hydrogen bonds link the mol­ecules into infinite zigzag chains extending along [100].

## Related literature

For the biological and pharmaceutical importance of quinazolines, see: Arfan *et al.* (2008 ▶); Bartroli *et al.* (1998 ▶); Kung *et al.* (1999 ▶); Mannschreck *et al.* (1984 ▶).
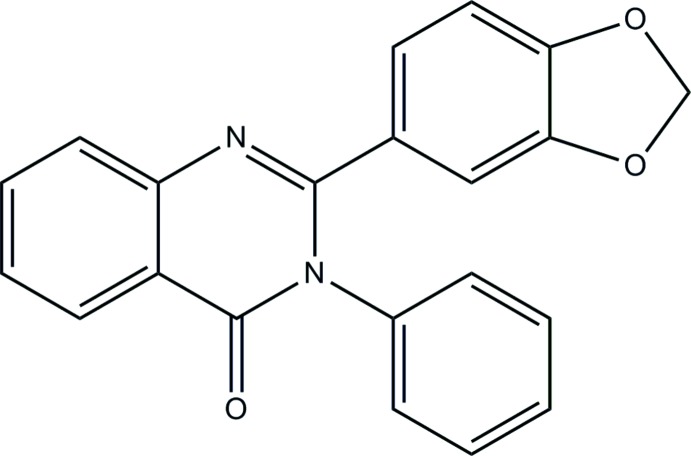


## Experimental

### 

#### Crystal data

C_21_H_14_N_2_O_3_
*M* *_r_* = 342.34
Monoclinic, 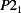

*a* = 8.984 (4) Å
*b* = 6.056 (3) Å
*c* = 15.248 (6) Å
β = 95.357 (6)°
*V* = 826.0 (6) Å^3^
*Z* = 2
Mo *K*α radiation
μ = 0.09 mm^−1^
*T* = 273 K
0.30 × 0.25 × 0.20 mm

#### Data collection

Bruker APEXII CCD area-detector diffractometer
9157 measured reflections
3751 independent reflections
3163 reflections with *I* > 2σ(*I*)
*R*_int_ = 0.023

#### Refinement

*R*[*F* ^2^ > 2σ(*F* ^2^)] = 0.040
*wR*(*F* ^2^) = 0.101
*S* = 1.04
3751 reflections
235 parameters
1 restraint
H-atom parameters constrained
Δρ_max_ = 0.16 e Å^−3^
Δρ_min_ = −0.20 e Å^−3^

### 

Data collection: *APEX2* (Bruker, 2009 ▶); cell refinement: *SAINT* (Bruker, 2009 ▶); data reduction: *SAINT*; program(s) used to solve structure: *SHELXS97* (Sheldrick, 2008 ▶); program(s) used to refine structure: *SHELXL97* (Sheldrick, 2008 ▶); molecular graphics: *PLATON* (Spek, 2009 ▶); software used to prepare material for publication: *SHELXL97*.

## Supplementary Material

Crystal structure: contains datablock(s) global, I. DOI: 10.1107/S1600536813016346/fk2072sup1.cif

Structure factors: contains datablock(s) I. DOI: 10.1107/S1600536813016346/fk2072Isup2.hkl

Supplementary material file. DOI: 10.1107/S1600536813016346/fk2072Isup3.cml

Additional supplementary materials:  crystallographic information; 3D view; checkCIF report

## Figures and Tables

**Table 1:** Hydrogen-bond geometry (Å, °)

*D*—H⋯*A*	*D*—H	H⋯*A*	*D*⋯*A*	*D*—H⋯*A*
C17—H17⋯O5^i^	0.93	2.37	3.185 (3)	146
C25—H23*A*⋯O5^ii^	0.97	2.49	3.416 (3)	160

Symmetry codes: (i) 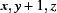; (ii) 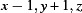.

**Fig. 1.**
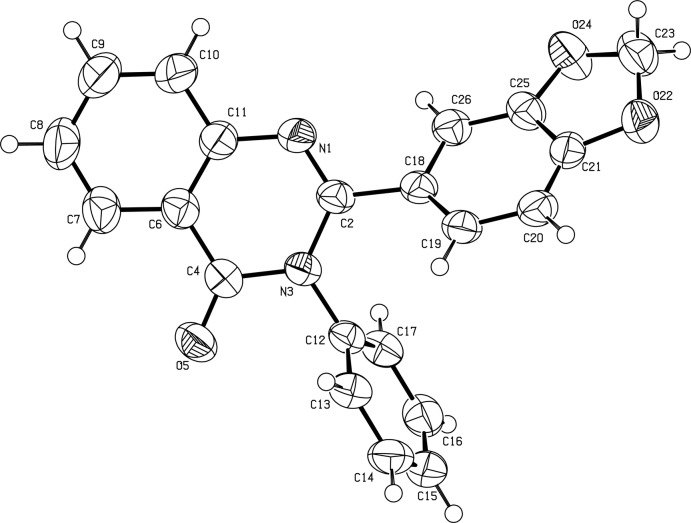
Molecular structure of the title compound with anisotropic displacement ellipsoids drawn at the 50% probability level.

**Fig. 2.**
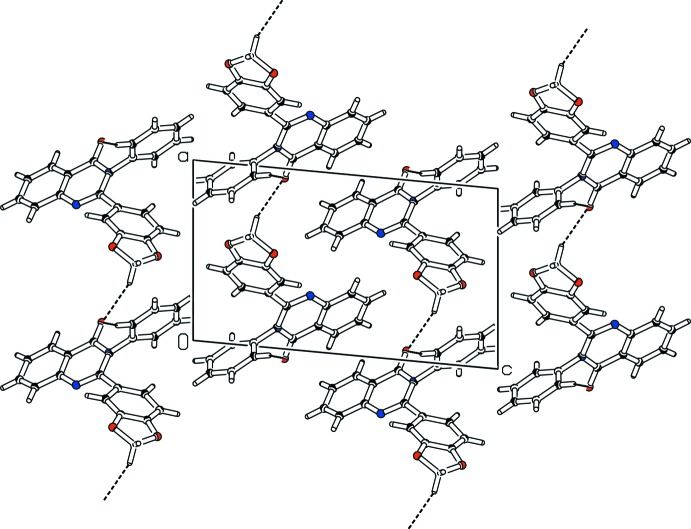
Packing diagram of the molecule viewed along b-axis. Intermolecular hydrogen bonding drawn as dotted lines.

## supplementary crystallographic information

### Comment

Quinazoline and their derivatives are an interesting class of heterocyclic compounds that have drawn much attention because of their biological and pharmaceutical activities; such as anti-bacterial (Kung *et al.*, 1999), antimicrobial (Arfan *et al.*, 2008) antifungal (Bartroli *et al.*, 1998) and anticonvulsant activities (Mannschreck *et al.*, 1984). In view of their importance, the crystal structure determination of the title compound was carried out and the results are presented herein.

In the molecular structure of the title compound (Fig. 1), the dihedral angles between the quinazoline moiety (N3–C4/C6–C11/N1–C2) and the phenyl ring (C12/C13/C14/C15/C16/C17) as well as the 1,3-benzodioxole ring (C18–C19/C20–C24/C25–C26) are 40.57 (9)° and 42.31 (11)°, respectively. The dihedral angle between the phenyl ring (C12/C13/C14/C15/C16/C17) and 1,3-benzodioxole ring (C18–C19/C20–C24/C25–C26) is 4.34 (10)°. The crystal packing exhibits intermolecular C—H···O interactions (Fig. 2) that link molecules into endless zig-zag chains extended along [100].

### Experimental

To a solution of 2-amino-*N*-phenylbenzamide (1 mmol) and benzo[*d*][1,3]dioxole-5-carbaldehyde (1 mmol) in ethyl acetate (2 ml) was added propyl phosphonic anhydride (1 mmol) and the reaction mixture was stirred for about 2 hrs at room temperature, then 2,3-Dichloro-5,6-dicyano-1,4-benzoquinone (DDQ) (1 mmol) was added and stirred for about 30 minutes. The reaction mixture was diluted with water and extracted to ethyl acetate and it was washed with 10% NaHCO3, water, brine solution and dried over anhydrous sodium sulfate and concentrated under reduced pressure to get a crude product which was recrystalized by slow evaporation in ethyl acetate at room temperature to get the title compound.

### Refinement

H atoms were placed at idealized positions and allowed to ride on their parent atoms with C–H distances in the range of 0.93 to 0.97 Å; *U*_iso_(H) = 1.2*U*_eq_(C).

The title compound crystallizes in the non-centrosymmetric space group P 2_1_; however, in the absence of significant anomalous scattering effects, the Flack parameter is essentially meaningless.

### Crystal data

**Table:**

C_21_H_14_N_2_O_3_	*F*(000) = 356
*M**_r_* = 342.34	*D*_x_ = 1.377 Mg m^−^^3^
Monoclinic, *P*2_1_	Mo *K*α radiation, λ = 0.71073 Å
Hall symbol: P 2yb	Cell parameters from 3751 reflections
*a* = 8.984 (4) Å	θ = 1.3–28.0°
*b* = 6.056 (3) Å	µ = 0.09 mm^−^^1^
*c* = 15.248 (6) Å	*T* = 273 K
β = 95.357 (6)°	Block, yellow
*V* = 826.0 (6) Å^3^	0.30 × 0.25 × 0.20 mm
*Z* = 2	

### Data collection

**Table:**

Bruker APEXII CCD area-detector diffractometer	*R*_int_ = 0.023
ω and φ scans	θ_max_ = 28.0°, θ_min_ = 1.3°
9157 measured reflections	*h* = −11→11
3751 independent reflections	*k* = −8→7
3163 reflections with *I* > 2σ(*I*)	*l* = −19→19

### Refinement

**Table:**

Refinement on *F*^2^	Primary atom site location: structure-invariant direct methods
Least-squares matrix: full	Secondary atom site location: difference Fourier map
*R*[*F*^2^ > 2σ(*F*^2^)] = 0.040	Hydrogen site location: inferred from neighbouring sites
*wR*(*F*^2^) = 0.101	H-atom parameters constrained
*S* = 1.04	*w* = 1/[σ^2^(*F*_o_^2^) + (0.0558*P*)^2^ + 0.0215*P*] where *P* = (*F*_o_^2^ + 2*F*_c_^2^)/3
3751 reflections	(Δ/σ)_max_ < 0.001
235 parameters	Δρ_max_ = 0.16 e Å^−^^3^
1 restraint	Δρ_min_ = −0.20 e Å^−^^3^

### Special details

**Table:**

Geometry. Bond distances, angles *etc*. have been calculated using the rounded fractional coordinates. All su's are estimated from the variances of the (full) variance-covariance matrix. The cell e.s.d.'s are taken into account in the estimation of distances, angles and torsion angles
Refinement. Refinement on *F*^2^ for ALL reflections except those flagged by the user for potential systematic errors. Weighted *R*-factors *wR* and all goodnesses of fit *S* are based on *F*^2^, conventional *R*-factors *R* are based on *F*, with *F* set to zero for negative *F*^2^. The observed criterion of *F*^2^ > σ(*F*^2^) is used only for calculating -*R*-factor-obs *etc*. and is not relevant to the choice of reflections for refinement. *R*-factors based on *F*^2^ are statistically about twice as large as those based on *F*, and *R*-factors based on ALL data will be even larger.

### Fractional atomic coordinates and isotropic or equivalent isotropic displacement parameters (Å^2^)

**Table:**

	*x*	*y*	*z*	*U*_iso_*/*U*_eq_	
O5	1.05179 (14)	0.2056 (2)	0.70071 (8)	0.0591 (4)	
O24	0.44816 (14)	1.2103 (3)	0.88187 (8)	0.0637 (4)	
O26	0.47712 (16)	1.2832 (2)	0.73629 (9)	0.0690 (5)	
N1	0.69195 (15)	0.5808 (3)	0.61645 (9)	0.0552 (5)	
N3	0.88645 (14)	0.4809 (2)	0.72424 (8)	0.0416 (4)	
C2	0.76074 (16)	0.6048 (3)	0.69362 (10)	0.0435 (5)	
C4	0.93930 (17)	0.3085 (3)	0.67377 (10)	0.0443 (5)	
C6	0.85398 (17)	0.2701 (3)	0.58978 (11)	0.0490 (5)	
C7	0.8883 (2)	0.0937 (4)	0.53638 (13)	0.0621 (7)	
C8	0.8062 (2)	0.0598 (5)	0.45769 (15)	0.0835 (9)	
C9	0.6918 (3)	0.2064 (6)	0.42996 (16)	0.1048 (12)	
C10	0.6579 (3)	0.3796 (6)	0.48092 (14)	0.0923 (9)	
C11	0.73748 (19)	0.4136 (4)	0.56322 (11)	0.0569 (6)	
C12	0.98242 (16)	0.5453 (3)	0.80117 (9)	0.0413 (5)	
C13	1.00197 (18)	0.4050 (3)	0.87253 (10)	0.0487 (5)	
C14	1.09842 (19)	0.4651 (4)	0.94460 (12)	0.0591 (6)	
C15	1.1735 (2)	0.6638 (4)	0.94456 (12)	0.0608 (7)	
C16	1.15312 (19)	0.8023 (3)	0.87348 (13)	0.0588 (6)	
C17	1.05718 (18)	0.7432 (3)	0.80046 (11)	0.0490 (5)	
C18	0.69538 (15)	0.7673 (3)	0.75203 (10)	0.0423 (5)	
C19	0.67842 (17)	0.7258 (3)	0.84001 (10)	0.0484 (5)	
C20	0.59554 (18)	0.8651 (3)	0.88969 (11)	0.0526 (6)	
C21	0.53344 (17)	1.0466 (3)	0.84817 (11)	0.0476 (5)	
C22	0.55001 (18)	1.0905 (3)	0.76149 (11)	0.0490 (5)	
C23	0.62930 (17)	0.9549 (3)	0.71145 (10)	0.0471 (5)	
C25	0.3916 (2)	1.3373 (4)	0.80727 (13)	0.0644 (7)	
H7	0.96700	−0.00050	0.55440	0.0750*	
H8	0.82650	−0.06040	0.42280	0.1000*	
H9	0.63780	0.18510	0.37560	0.1260*	
H10	0.58160	0.47610	0.46110	0.1110*	
H13	0.95090	0.27130	0.87220	0.0580*	
H14	1.11260	0.37170	0.99310	0.0710*	
H15	1.23820	0.70390	0.99310	0.0730*	
H16	1.20370	0.93640	0.87410	0.0710*	
H17	1.04380	0.83620	0.75180	0.0590*	
H19	0.72360	0.60150	0.86660	0.0580*	
H20	0.58330	0.83540	0.94840	0.0630*	
H23A	0.28690	1.30300	0.79170	0.0770*	
H23B	0.40020	1.49370	0.82050	0.0770*	
H26	0.63900	0.98610	0.65250	0.0570*	

### Atomic displacement parameters (Å^2^)

**Table:**

	*U*^11^	*U*^22^	*U*^33^	*U*^12^	*U*^13^	*U*^23^
O5	0.0584 (7)	0.0551 (7)	0.0635 (7)	0.0186 (6)	0.0045 (6)	0.0065 (6)
O24	0.0545 (7)	0.0728 (9)	0.0645 (7)	0.0097 (7)	0.0101 (6)	−0.0164 (7)
O26	0.0705 (9)	0.0601 (8)	0.0795 (8)	0.0209 (7)	0.0228 (7)	0.0092 (7)
N1	0.0408 (7)	0.0766 (11)	0.0470 (7)	0.0126 (8)	−0.0016 (6)	−0.0096 (8)
N3	0.0370 (6)	0.0445 (7)	0.0430 (6)	0.0033 (6)	0.0025 (5)	0.0029 (6)
C2	0.0338 (7)	0.0526 (10)	0.0441 (8)	0.0033 (7)	0.0035 (6)	0.0023 (7)
C4	0.0430 (8)	0.0414 (9)	0.0497 (9)	0.0006 (7)	0.0105 (7)	0.0053 (7)
C6	0.0392 (8)	0.0560 (11)	0.0533 (9)	−0.0022 (8)	0.0125 (7)	−0.0040 (8)
C7	0.0522 (10)	0.0674 (13)	0.0688 (11)	−0.0017 (9)	0.0163 (9)	−0.0163 (10)
C8	0.0590 (12)	0.108 (2)	0.0847 (14)	0.0021 (14)	0.0134 (11)	−0.0491 (15)
C9	0.0626 (13)	0.169 (3)	0.0792 (14)	0.0229 (17)	−0.0125 (11)	−0.0633 (17)
C10	0.0616 (12)	0.142 (2)	0.0694 (13)	0.0328 (15)	−0.0145 (10)	−0.0415 (15)
C11	0.0403 (8)	0.0769 (13)	0.0534 (9)	0.0035 (9)	0.0033 (7)	−0.0150 (9)
C12	0.0325 (7)	0.0455 (9)	0.0458 (8)	0.0052 (7)	0.0040 (6)	0.0019 (7)
C13	0.0408 (8)	0.0539 (10)	0.0514 (9)	0.0014 (8)	0.0039 (6)	0.0096 (8)
C14	0.0481 (9)	0.0804 (14)	0.0480 (9)	0.0125 (10)	0.0005 (7)	0.0101 (9)
C15	0.0433 (9)	0.0767 (15)	0.0607 (11)	0.0098 (9)	−0.0041 (8)	−0.0119 (10)
C16	0.0437 (9)	0.0525 (10)	0.0799 (12)	−0.0023 (9)	0.0036 (8)	−0.0120 (10)
C17	0.0425 (8)	0.0440 (9)	0.0601 (10)	0.0036 (7)	0.0026 (7)	0.0071 (8)
C18	0.0303 (7)	0.0518 (9)	0.0444 (8)	0.0002 (7)	0.0013 (6)	−0.0016 (7)
C19	0.0390 (8)	0.0597 (10)	0.0458 (8)	0.0077 (8)	0.0001 (6)	0.0028 (8)
C20	0.0425 (8)	0.0740 (13)	0.0413 (8)	0.0036 (9)	0.0035 (6)	−0.0016 (8)
C21	0.0345 (7)	0.0593 (11)	0.0487 (8)	−0.0016 (7)	0.0031 (6)	−0.0131 (8)
C22	0.0395 (8)	0.0478 (9)	0.0599 (10)	0.0020 (8)	0.0064 (7)	−0.0001 (8)
C23	0.0395 (8)	0.0580 (10)	0.0443 (8)	0.0009 (8)	0.0063 (6)	0.0022 (8)
C25	0.0517 (10)	0.0643 (12)	0.0785 (12)	0.0096 (9)	0.0130 (9)	−0.0074 (10)

### Geometric parameters (Å, º)

**Table:**

O5—C4	1.225 (2)	C15—C16	1.369 (3)
O24—C21	1.381 (2)	C16—C17	1.390 (3)
O24—C25	1.427 (3)	C18—C19	1.387 (2)
O26—C22	1.375 (2)	C18—C23	1.399 (3)
O26—C25	1.423 (2)	C19—C20	1.395 (2)
N1—C2	1.285 (2)	C20—C21	1.362 (3)
N1—C11	1.383 (3)	C21—C22	1.370 (2)
N3—C2	1.399 (2)	C22—C23	1.366 (2)
N3—C4	1.406 (2)	C7—H7	0.9300
N3—C12	1.443 (2)	C8—H8	0.9300
C2—C18	1.485 (2)	C9—H9	0.9300
C4—C6	1.449 (2)	C10—H10	0.9300
C6—C7	1.395 (3)	C13—H13	0.9300
C6—C11	1.391 (3)	C14—H14	0.9300
C7—C8	1.364 (3)	C15—H15	0.9300
C8—C9	1.394 (4)	C16—H16	0.9300
C9—C10	1.357 (5)	C17—H17	0.9300
C10—C11	1.400 (3)	C19—H19	0.9300
C12—C13	1.379 (2)	C20—H20	0.9300
C12—C17	1.374 (3)	C23—H26	0.9300
C13—C14	1.383 (3)	C25—H23A	0.9700
C14—C15	1.380 (3)	C25—H23B	0.9700
			
C21—O24—C25	105.01 (14)	O24—C21—C22	109.49 (15)
C22—O26—C25	105.05 (14)	C20—C21—C22	121.91 (16)
C2—N1—C11	118.47 (15)	O26—C22—C21	110.03 (15)
C2—N3—C4	121.26 (13)	O26—C22—C23	127.86 (15)
C2—N3—C12	121.91 (13)	C21—C22—C23	122.12 (16)
C4—N3—C12	116.04 (12)	C18—C23—C22	117.59 (14)
N1—C2—N3	123.30 (15)	O24—C25—O26	107.75 (16)
N1—C2—C18	116.16 (14)	C6—C7—H7	120.00
N3—C2—C18	120.49 (13)	C8—C7—H7	120.00
O5—C4—N3	120.30 (14)	C7—C8—H8	120.00
O5—C4—C6	124.52 (16)	C9—C8—H8	120.00
N3—C4—C6	115.17 (14)	C8—C9—H9	119.00
C4—C6—C7	120.76 (16)	C10—C9—H9	119.00
C4—C6—C11	118.60 (16)	C9—C10—H10	120.00
C7—C6—C11	120.64 (16)	C11—C10—H10	120.00
C6—C7—C8	119.9 (2)	C12—C13—H13	120.00
C7—C8—C9	119.6 (2)	C14—C13—H13	120.00
C8—C9—C10	121.2 (2)	C13—C14—H14	120.00
C9—C10—C11	120.3 (3)	C15—C14—H14	120.00
N1—C11—C6	122.83 (15)	C14—C15—H15	120.00
N1—C11—C10	118.6 (2)	C16—C15—H15	120.00
C6—C11—C10	118.4 (2)	C15—C16—H16	120.00
N3—C12—C13	119.83 (15)	C17—C16—H16	120.00
N3—C12—C17	118.99 (14)	C12—C17—H17	120.00
C13—C12—C17	121.15 (14)	C16—C17—H17	120.00
C12—C13—C14	119.28 (17)	C18—C19—H19	119.00
C13—C14—C15	119.95 (18)	C20—C19—H19	119.00
C14—C15—C16	120.36 (17)	C19—C20—H20	122.00
C15—C16—C17	120.23 (17)	C21—C20—H20	122.00
C12—C17—C16	119.03 (16)	C18—C23—H26	121.00
C2—C18—C19	123.05 (16)	C22—C23—H26	121.00
C2—C18—C23	116.63 (14)	O24—C25—H23A	110.00
C19—C18—C23	119.63 (15)	O24—C25—H23B	110.00
C18—C19—C20	121.88 (16)	O26—C25—H23A	110.00
C19—C20—C21	116.88 (15)	O26—C25—H23B	110.00
O24—C21—C20	128.60 (15)	H23A—C25—H23B	108.00
			
C25—O24—C21—C20	−170.35 (18)	C11—C6—C7—C8	−0.6 (3)
C25—O24—C21—C22	9.67 (19)	C4—C6—C11—N1	−5.3 (3)
C21—O24—C25—O26	−15.83 (19)	C4—C6—C11—C10	178.3 (2)
C25—O26—C22—C21	−10.09 (19)	C7—C6—C11—N1	175.08 (18)
C25—O26—C22—C23	170.00 (18)	C7—C6—C11—C10	−1.4 (3)
C22—O26—C25—O24	15.97 (19)	C6—C7—C8—C9	2.2 (3)
C11—N1—C2—N3	4.8 (3)	C7—C8—C9—C10	−1.7 (4)
C11—N1—C2—C18	−172.49 (16)	C8—C9—C10—C11	−0.4 (4)
C2—N1—C11—C6	0.3 (3)	C9—C10—C11—N1	−174.8 (2)
C2—N1—C11—C10	176.7 (2)	C9—C10—C11—C6	1.9 (4)
C4—N3—C2—N1	−4.8 (2)	N3—C12—C13—C14	177.68 (15)
C4—N3—C2—C18	172.43 (14)	C17—C12—C13—C14	0.1 (3)
C12—N3—C2—N1	164.67 (16)	N3—C12—C17—C16	−178.07 (15)
C12—N3—C2—C18	−18.1 (2)	C13—C12—C17—C16	−0.5 (2)
C2—N3—C4—O5	178.13 (15)	C12—C13—C14—C15	0.1 (3)
C2—N3—C4—C6	−0.4 (2)	C13—C14—C15—C16	0.0 (3)
C12—N3—C4—O5	8.1 (2)	C14—C15—C16—C17	−0.4 (3)
C12—N3—C4—C6	−170.47 (14)	C15—C16—C17—C12	0.6 (3)
C2—N3—C12—C13	120.18 (17)	C2—C18—C19—C20	−169.50 (15)
C2—N3—C12—C17	−62.2 (2)	C23—C18—C19—C20	0.7 (2)
C4—N3—C12—C13	−69.84 (18)	C2—C18—C23—C22	170.86 (14)
C4—N3—C12—C17	107.81 (17)	C19—C18—C23—C22	0.1 (2)
N1—C2—C18—C19	135.61 (17)	C18—C19—C20—C21	−1.0 (2)
N1—C2—C18—C23	−34.8 (2)	C19—C20—C21—O24	−179.44 (16)
N3—C2—C18—C19	−41.8 (2)	C19—C20—C21—C22	0.5 (2)
N3—C2—C18—C23	147.76 (15)	O24—C21—C22—O26	0.25 (19)
O5—C4—C6—C7	6.2 (3)	O24—C21—C22—C23	−179.83 (16)
O5—C4—C6—C11	−173.46 (17)	C20—C21—C22—O26	−179.74 (15)
N3—C4—C6—C7	−175.32 (16)	C20—C21—C22—C23	0.2 (3)
N3—C4—C6—C11	5.0 (2)	O26—C22—C23—C18	179.41 (16)
C4—C6—C7—C8	179.72 (19)	C21—C22—C23—C18	−0.5 (2)

### Hydrogen-bond geometry (Å, º)

**Table:**

*D*—H···*A*	*D*—H	H···*A*	*D*···*A*	*D*—H···*A*
C17—H17···O5^i^	0.93	2.37	3.185 (3)	146
C25—H23*A*···O5^ii^	0.97	2.49	3.416 (3)	160

Symmetry codes: (i) *x*, *y*+1, *z*; (ii) *x*−1, *y*+1, *z*.
